# Recovery of data from perfectly twinned virus crystals revisited

**DOI:** 10.1107/S2059798316007117

**Published:** 2016-05-25

**Authors:** Helen Mary Ginn, David Ian Stuart

**Affiliations:** a Division of Structural Biology, Wellcome Trust Centre for Human Genetics, Roosevelt Drive, Oxford OX3 7BN, England; b Diamond House, Harwell Science and Innovation Campus, Fermi Avenue, Didcot OX11 0QX, England

**Keywords:** perfect merohedral twinning, noncrystallographic symmetry, virus crystals

## Abstract

Perfect merohedral twinning of crystals is not uncommon and complicates structural analysis. An iterative method for the deconvolution of data from perfectly merohedrally twinned crystals in the presence of noncrystallographic symmetry (NCS) has been reimplemented. It is shown that the method recovers the data effectively using test data, and an independent metric of success, based on special classes of reflections that are unaffected by the twin operator, is now provided. The method was applied to a real problem with fivefold NCS and rather poor-quality diffraction data, and it was found that even in these circumstances the method appears to recover most of the information. The software has been made available in a form that can be applied to other crystal systems.

## Abstract 2

An iterative method of map recovery for perfectly merohedrally twinned crystals in the presence of noncrystallographic symmetry is reimplemented and released and an independent metric of success is provided.

## 1. Introduction   

Biological crystals are occasionally, but not uncommonly, subject to perfect or imperfect merohedral twinning (Yeates, 1997 ▸; Yeates & Fam, 1999 ▸), where unit cells or mosaic domains are randomly distributed into two or more orientations without affecting the crystal lattice. This is particularly common in virus-capsid crystallography, where spherical capsids can rotate without significantly altering the minimal crystal contacts (Lerch *et al.*, 2009 ▸). For some crystal systems, twinning can be minimized or avoided by altering the concentration of nuclei for crystallization (Chayen & Saridakis, 2008 ▸) or deliberately choosing crystals that grow at a slower rate (Borshchevskiy *et al.*, 2009 ▸). When the merohedral twinning fraction is measurably below 0.5, data recovery is comparatively easier and quite frequently allows structure solution by *de novo* methods. For molecular-replacement solutions there are a large number of examples (Breyer *et al.*, 1999 ▸; Igarashi *et al.*, 1997 ▸; Carr *et al.*, 1996 ▸; Luecke *et al.*, 1998 ▸; Chandra *et al.*, 1999 ▸; Contreras-Martel *et al.*, 2001 ▸). For anomalous phasing, notable examples include interleukin-1 (Rudolph *et al.*, 2003 ▸) and a selenomethionine variant of the capsid-stabilizing protein of bacteriophage λ, gpD (Yang *et al.*, 2000 ▸), which were both solved by multiwavelength anomalous dispersion (MAD). Twinned crystals of bilirubin oxidase with a twin fraction of 0.487 were solved by SAD (Mizutani *et al.*, 2010 ▸). However, perfect merohedral twinning is often more challenging to overcome, and most commonly requires molecular replacement to solve the structure (Chandra *et al.*, 1999 ▸; Redinbo & Yeates, 1993 ▸; Lea & Stuart, 1995 ▸). However, the gpD structure has been solved by SAD, where the data were averaged to emulate a twinning fraction of 0.5 (Dauter, 2003 ▸). Twinning presents itself as a higher symmetry space group and may be more difficult to detect immediately if analysis of the crystal-packing density is not unambiguous. However, it causes an enrichment of mid-intensity reflections owing to the superposition of the two crystal orientations, where combinations of two low-intensity or two high-intensity reflections are less common. In fact, it is common for proteins to be submitted to the PDB with their partially twinned nature going unnoticed (Lebedev *et al.*, 2006 ▸). Programs such as *TRUNCATE*, which is part of the *CCP*4 suite, now test for this distorted intensity distribution as standard (Winn *et al.*, 2011 ▸).

*Foot-and-mouth disease virus* (FMDV) crystals of the O_1_M variant form perfectly merohedrally twinned crystals similar to those of the G67 variant caused by a 90° difference in the orientation of 50% of the virions in the crystal. The previously solved structures of the O_1_BFS (PDB entry 1bbt) and O_1_K variants lack the point mutations at residues 72–74 that were proposed to give rise to twinning, and they therefore form untwinned crystals in space group *I*23 (Acharya *et al.*, 1989 ▸; Lea *et al.*, 1995 ▸). In *I*23, ignoring anomalous differences, such perfect twinning makes reflections (*h*, *k*, *l*) and (*k*, *h*, *l*) equivalent, creating pseudo-fourfold symmetry that emulates the symmetry of the *I*432 space group. In this case, this can be distinguished from a true *I*432 space group as icosahedral viruses do not possess fourfold symmetry and the unit-cell dimensions only permit a single virion in the unit cell. Reflections where *h* = *k* are unaffected by twinning (here referred to as ‘singlet’ reflections). Note that depending on the definition of the asymmetric unit, this can also include *h* = *l* and *k* = *l*. Twinning in the G67 variant has been shown to occur at the level of mosaic blocks as the paired structure factors correlate most strongly with the mean intensity of untwinned O_1_BFS structure-factor twin pairs rather than the vector mean (Lea & Stuart, 1995 ▸). Importantly, the icosahedral virus capsid pentamers cannot be part of the crystallographic symmetry, and are therefore present in the NCS operations, which is key to this study.

We aimed to recover a set of untwinned structure factors from these perfectly twinned data, using a method that has been described previously to deconvolute similar data sets (Lea & Stuart, 1995 ▸). This is an unusual procedure, as it is said conventionally that untwinned intensities cannot be recovered from perfectly twinned data sets, unlike those that have a twinning fraction of less than 50%. The procedure is designed to obtain a set of untwinned structure factors that are consistent with the *F*_obs_ measurements, while producing an electron-density map that obeys the known fivefold NCS. In other words, after recovery of the untwinned intensities, the average of the intensities of each twin pair of reflections would be equal to the original twinned intensity. In order to generate the untwinned intensities, the intensities must be biased towards their true values. If a data set had no NCS, it would not be possible to bias the intensities enough to recover the untwinned structure factors. However, with fivefold NCS, which breaks the symmetry produced by the 90° rotation twinning operation, it is possible to bias the original intensities towards their untwinned values and recover the untwinned intensities over several iterative cycles of refinement. Fivefold averaging across one axis causes constructive interference of signal for one orientation of the virion, whereas the 90°-related virions do not possess this symmetry and average out to noise. After this, one must ensure that paired reflection intensities respect the twinning law: this is performed by rescaling individual pairs of reflections such that the average of the corresponding intensities matches that of the original twinned intensities. This is followed by additional cycles of NCS averaging and application of the twinning law until the procedure converges.

**Figure 1:**
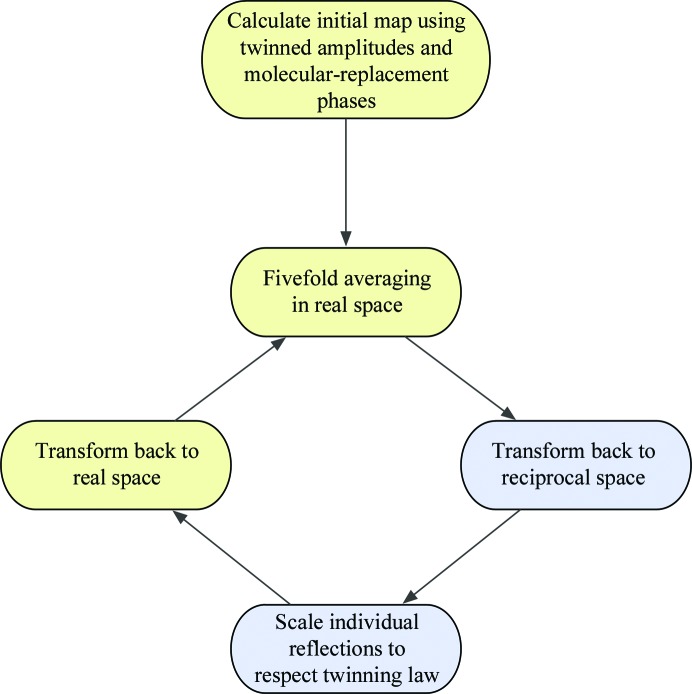
Strategy for deconvolution of twinned data sets; yellow boxes within the cycle are carried out in real space and blue boxes are carried out in reciprocal space. The cycle is typically executed 20 times, at which point convergence has been achieved.

We have made the source code available for others to use, and a summary of the method (iterative cycles of NCS averaging, application of the twinning law and rescaling of the structure factors) is provided in Fig. 1 ▸. As a control, a set of structure factors were generated from FMDV O_1_BFS coordinates. These intensities came from naturally untwinned crystals that were artifically ‘retwinned’ by averaging the (*h*, *k*, *l*) and (*k*, *h*, *l*) intensities. This study reimplements the method and seeks to validate the procedure using these ‘retwinned’ O_1_BFS structure factors as a control and assess the quality of recovery from twinned O_1_M data in a more rigorous fashion than previously attempted. The experimental details of crystal preparation and the derived structure are reported in another paper (Kotecha *et al.*, 2015 ▸).

## 2. Materials and methods   

### 2.1. Artificial twinning of O_1_BFS reflections   

Untwinned O_1_BFS structure factors were obtained from the PDB (entry 1bbt). To ‘retwin’ the data, intensities were averaged between the twin reflection pairs. To reduce the quality of the O_1_BFS phases to be similar to the quality of the O_1_M phases (derived as described below), rigid-body refinement, positional minimization and *B*-factor refinement was performed using retwinned O_1_BFS amplitudes and the atomic coordinates of O_1_BFS in *CNS* v.1.3 (Brunger, 2007 ▸).

### 2.2. Generation of preliminary phases   

The intensities for the O_1_M data set were scaled and merged in space group *I*432 and expanded to space group *I*23. Preliminary phases for O_1_M were generated in *CNS* by rigid-body refinement using the atomic coordinates of O_1_BFS and the twinned amplitudes from the O_1_M data. The model was further refined by minimization and *B*-factor refinement.

### 2.3. NCS averaging   

A solvent-flattening envelope was generated for electron-density maps by setting the interior and exterior of the protein capsid to a density of 0 using the *General Averaging Program* (*GAP*; Grimes *et al.*, 1998 ▸). Electron-density maps were averaged using the envelope and symmetry operators representing the fivefold NCS present in these data. The calculated data were transformed back to reciprocal space for scaling.

### 2.4. Resolution-shell scaling   

Reflections were categorized into 20 resolution shells, each containing a similar number of data. All calculated amplitudes were scaled to observed amplitudes using a scale factor *F*_obs_/*F*_calc_ generated using only singlet reflections within each resolution shell, as these are not affected by twinning. The number of such reflections was between 89 and 360, so the scale factors were likely to be statistically reliable.

### 2.5. Twinning-law scaling   

A scale factor *k* was generated and applied to each related pair of reflections in order to generate calculated amplitudes that are consistent with the observed amplitudes in the twinned data set according to (1), while keeping the ratio between the pair of amplitudes the same:
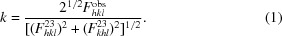
Except for the final iteration, singlet data were adjusted to (2*F*_obs_ − *F*_calc_) before scaling rather than setting them equal to their known values. In the last round of refinement, singlet reflections were set to the original amplitudes from the twinned data set. Structure factors were transformed to real space if sequential rounds of NCS averaging and scaling were required.

## 3. Results   

**Figure 2:**
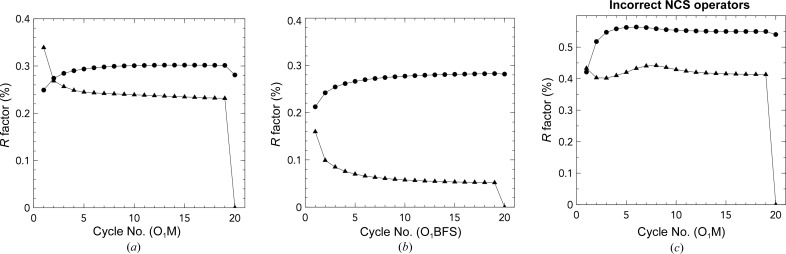
*R*_singlets_ (triangles) and *R*_all_ (circles) values for deconvolution of the twinned O_1_M data set (*a*) and the artificially twinned O_1_BFS data set (*b*). *R*_all_ values diverge while *R*_singlets_ values converge; singlet reflections are not affected by twinning operators. If NCS operators are rotated by 90° in the *x* axis and deconvolution is attempted (*c*), the *R* factors do not show signs of success, as expected.

Reflections for O_1_M and artifically twinned O_1_BFS were transformed into real space. These electron-density maps were averaged using fivefold NCS and scaled according to resolution shell using only singlet reflections for a total of 20 cycles. *R* factors and correlation coefficients were measured between observed twinned data and partially detwinned data, for both the whole set of reflections (*R*_all_, CC_all_) and the singlet subset (*R*_singlets_, CC_singlets_), at each stage of the cycle (*R* factors are shown in Fig. 2 ▸, including the result from incorrect NCS operators). The singlet reflections are treated specially, rather than setting them equal to the amplitudes in the twinned data set: they are only scaled globally. This allows them to be used as a measure of success by tracking their agreement with the original amplitudes over several rounds of fivefold NCS averaging, as they are unaffected by twinning.

The O_1_BFS data set is of high quality, with a standard error (σ_obs_/*F*_obs_) of 4.2%, reflecting the excellent diffraction from these crystals. *R*_all_ for the O_1_BFS control shows sequential divergence between the twinned and deconvoluted data sets, reaching a maximum of 28.3% and a correlation coefficient (CC_all_) of 0.591. *R*_singlets_ improves from 15.9 to 5.8%, showing excellent prediction of singlet values by the deconvoluted data set. This is corroborated by the maximum CC_singlets_ value of 0.978. The *R* factor comparing all of the original untwinned O_1_BFS amplitudes and the deconvoluted amplitudes shows strong agreement at 9.3%. The algorithms used to reassign negative reflection intensities during data processing of the diffraction patterns (French & Wilson, 1978 ▸) tend to skew the weakest original amplitudes towards slightly higher calculated values, which is corrected post-deconvolution. This suggests that the original amplitudes can be largely recovered to the limitations of the standard error of the untwinned amplitudes.

The phases generated for the twinned O_1_M data set were of poor quality and resulted in a poor preliminary *R* factor of 38.6%, as shown in Table 1 ▸. *R*_all_ for O_1_M closely follows that of the O_1_BFS data, reaching a maximum of 28.8% with a CC_all_ of 0.715. The *R*_singlets_ shows that the calculated singlet reflections more closely match the observed data at a final converged value of 21.2% and a CC_singlets_ value of 0.941 before the final cycle. The major source of error in the higher *R*_singlets_ and lower CC_singlets_ values compared with the O_1_BFS data is likely to be the poorer crystal quality and diffraction; the high standard error (σ_obs_/*F*_obs_) for the O_1_M data set is 15.4% for all reflections. Other sources of error include the reassignment of negative intensities and the use the O_1_BFS coordinates to generate phases, which will be of poorer quality. However, the drop in *R*_singlets_ to a final value that is within 6% discrepancy of one standard deviation suggests that the near-maximal recovery of the detwinned amplitudes has been achieved compared with the control, despite the poorer quality of the data set.

**Figure 3:**
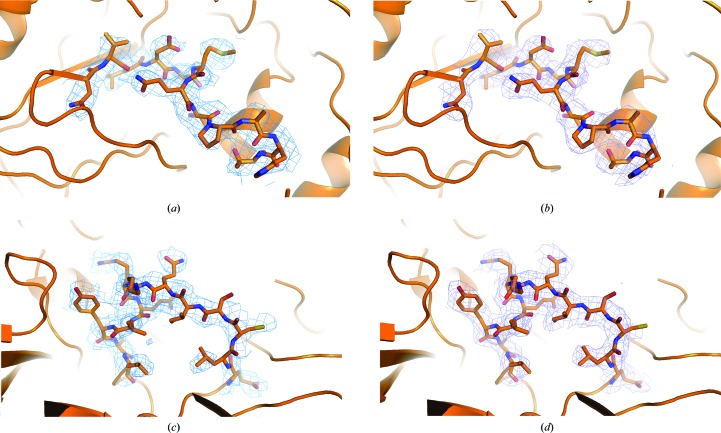
Density around Met54 of VP3 in the O_1_Manisa variant, showing breakage of main-chain density in the twinned structure (*a*) and recovery of main-chain density in the detwinned structure (*b*). Similarly, twinned density (*c*) and detwinned density (*d*) is shown around residue Gln133 of VP1. Phases were derived from refinement with *PHENIX* in both maps. Density is drawn at a σ of 1.0.

**Figure 4:**
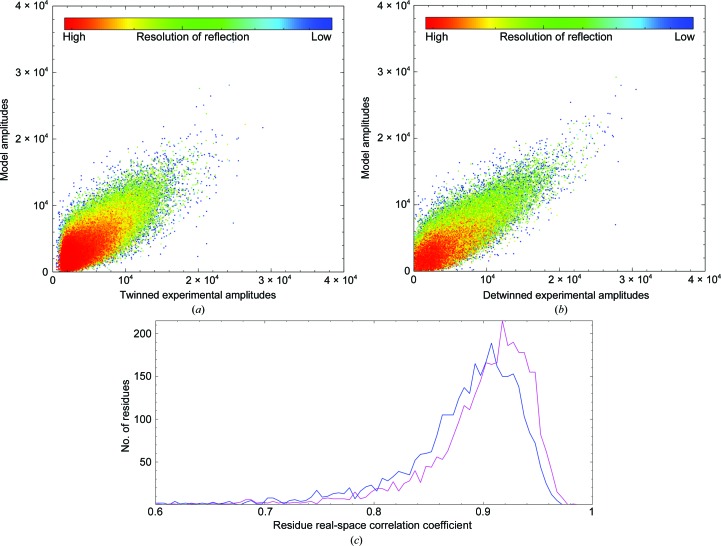
Amplitude plots between *F*_obs_ and *F*_calc_ for the twinned data sets (*a*) and detwinned data sets (*b*). The frequency of real-space correlation coefficient per residue is plotted in (*c*), where the blue line is derived from the map from refinement of the model against the twinned amplitude (the mean correlation is 0.87) and the magenta line is derived similarly from the detwinned amplitudes (the mean correlation is 0.89).

The improvement in density is seen immediately after deconvolution, without any need for extensive structure refinement. After deconvolution the structure can be refined and generates good-quality electron-density maps in *PHENIX* (an illustrative example is given in Fig. 3 ▸). These refined coordinates can be refined against the twinned data set as well and the density compared. It is apparent from the shape of the *F*_obs_ to *F*_calc_ distribution from *PHENIX* (Adams *et al.*, 2010 ▸) that the twinned data have a distorted distribution of *F*_obs_ values, with an enrichment of mid-intensity reflections that match a wide range of *F*_calc_ values. This is reflected in the CC_work_ increasing from 76.8% (twinned) to 81.5% (detwinned). The real-space correlation coefficient between individual residues increases from 87.7% against the twinned data to 89.4% against the detwinned data across each of the five NCS copies of 660 residues and is clearly elevated throughout the sequence of the protein chains (Fig. 4 ▸).

## 4. Conclusion   

The data analysis suggests that the deconvolution of twinned crystals with rotational NCS, which is distinct from the symmetry of the twinning operators, is successful. The control data set used here also suggests that the error can be reduced to within 6% of the error already present during data collection. The success of the deconvolution process can be measured by separately processing and tracking the *R* factor for singlet reflections only, and is verified visually by comparing the electron density. Furthermore, this method will be highly applicable to other virus crystal structures that possess high rotational NCS and a high propensity for twinning owing to their pseudo-spherical nature, as well as other twinned structures that exhibit similar NCS and twinning-operator relationships. This could be applied to the six point groups that support true merohedral twinning (Yeates, 1997 ▸). Tables of space groups that can lead to this problem, point groups and possible twin operators have been discussed (Chandra *et al.*, 1999 ▸). The source code for solving hemihedral twinning, written primarily in C++, is available along with an example structure and script (http://github.com/helenginn/deconvolute). It requires the CCP4 tools to be installed, but provides the other external Fortran tools required to run the program. Compilation has been tested on the *GCC* compiler v.4.4.7.

*Note added in proof*. Following the submission of this paper, a study also dealing with the use of NCS to aid in the handling of perfectly twinned diffraction data was published by Sabin & Plevka (2016 ▸).

## Figures and Tables

**Table 1:** Preliminary statistics for the O_1_M reflection data set prior to the data-recovery algorithm

Resolution range (Å)	43.63–2.90
Space group	*I*23
Unit-cell parameters (Å)	*a* = *b* = *c* = 344.08
No. of unique reflections	69889
Multiplicity	2.1
*R*_merge_ (%)	30.9
Completeness (outer shell) (%)	92.3 (77.4)
*R*_work_, pre-deconvolution (%)	38.6
*R*_work_, post-deconvolution (%)	37.5
*R*_work_, post-model refinement (%)	33.9
